# The Effects of Augmented Reality Visual Cues on Turning in Place in Parkinson's Disease Patients With Freezing of Gait

**DOI:** 10.3389/fneur.2020.00185

**Published:** 2020-03-24

**Authors:** Sabine Janssen, Jaap de Ruyter van Steveninck, Hizirwan S. Salim, Helena M. Cockx, Bastiaan R. Bloem, Tjitske Heida, Richard J. A. van Wezel

**Affiliations:** ^1^Biomedical Signals and Systems Group, MIRA Institute for Biomedical Technology and Technical Medicine, University of Twente, Enschede, Netherlands; ^2^Radboud University Medical Centre, Donders Institute for Brain, Cognition and Behaviour, Department of Neurology, Centre of Expertise for Parkinson & Movement Disorders, Nijmegen, Netherlands; ^3^Department of Biophysics, Donders Institute for Brain, Cognition and Behaviour, Radboud University, Nijmegen, Netherlands

**Keywords:** Parkinson Disease, cues, augmented reality, freezing of gait, treatment, rehabilitation

## Abstract

**Background:** Turning in place is particularly bothersome for patients with Parkinson's disease (PD) experiencing freezing of gait (FOG). Cues designed to enforce goal-directed turning are not yet available.

**Objectives:** Assess whether augmented reality (AR) visual cues improve FOG and turning in place in PD patients with FOG.

**Methods:** Sixteen PD patients with FOG performed a series of 180° turns under an experimental condition with AR visual cues displayed through a HoloLens and two control conditions (one consisting of auditory cues and one without any cues). FOG episodes were annotated by two independent raters from video recordings. Motion data were measured with 17 inertial measurement units for calculating axial kinematics, scaling, and timing of turning.

**Results:** AR visual cues did not reduce the percent time frozen (*p* = 0.73) or the number (*p* = 0.73) and duration (*p* = 0.78) of FOG episodes compared to the control condition without cues. All FOG parameters were higher with AR visual cues than with auditory cues [percent time frozen (*p* = 0.01), number (*p* = 0.02), and duration (*p* = 0.007) of FOG episodes]. The AR visual cues did reduce the peak angular velocity (visual vs. uncued *p* = 0.03; visual vs. auditory *p* = 0.02) and step height (visual vs. uncued *p* = 0.02; visual vs. auditory *p* = 0.007), and increased the step height coefficient of variation (visual vs. uncued *p* = 0.04; visual vs. auditory *p* = 0.01) and time to maximum head–pelvis separation (visual vs. uncued *p* = 0.02; visual vs. auditory *p* = 0.005), compared to both control conditions.

**Conclusions:** The AR visual cues in this study did not reduce FOG, and worsened some measures of axial kinematics, and turn scaling and timing. Stimulating goal-directed turning might, by itself, be insufficient to reduce FOG and improve turning performance.

**Trial Registration:** This study was registered in the Dutch trial registry (NTR6409; 2017-02-16).

## Introduction

Turning in place is an inevitable part of daily life mobility that can be particularly bothersome to patients with Parkinson's disease (PD). This is especially true for those patients who experience freezing of gait (FOG), a disturbing motor symptom defined as a “brief, episodic absence or marked reduction of forward progression of the feet despite the intention to walk” (1). Turning is the most common trigger to elicit FOG (2, 3). Both FOG and turns increase the risks of falling and fall-related injuries (4, 5).

Compared to healthy controls, PD patients perform turns more slowly (6–8), with a wider turning arc (8), shorter step length (8, 9), higher step count (6, 10), stronger coupling between head and trunk rotation (i.e., turning “en bloc”) (10), and less medial shifting of the center of mass (COM) (11). In PD patients with FOG, turn time, cadence, and head–trunk coupling are increased more than in patients without FOG (12, 13). The observation that the head–pelvis sequence (meaning that rotation of the head precedes the trunk) is delayed and reduced—or even absent—in trials with FOG (14) suggests a, not necessarily causal, relationship between head–trunk coupling and FOG. Furthermore, prior to a FOG episode, the COM deviation toward the inner leg is reduced compared to uninterrupted turning (7). This impaired weight shifting toward the inner leg might hinder unloading of the outer leg, thereby disrupting the normal stepping sequence and triggering FOG (7).

During regular straight walking, external cues can alleviate FOG and restore spatiotemporal gait deficits (15). One plausible working mechanism is that cues shift motor control from a habitual to a goal-directed mode of control, redirecting neural processing to less-affected neural circuits (16). The cueing strategies currently used specifically for turning (8, 17–21) apply a different strategy; i.e., they all provide an external timing to which steps can be synchronized to, but lack cues designed to enforce goal-directed movements. Providing a visual goal to turn toward possibly increases head–pelvis dissociation, restores COM shifting, and reduces FOG. Augmented reality (AR) displayed through smart glasses is particularly suited to provide interactive visual cues invoking goal-directed turning. Whether such cues are effective in reducing FOG during turning, and whether this is mediated by an effect on head–pelvis separation and medial COM shifting, is unknown.

This study aimed to assess whether AR visual cues could improve FOG and performance during turning in place in PD patients with FOG. Our primary objective was to assess whether AR visual cues influenced FOG severity compared to control conditions (no cues; and a conventional metronome). Our secondary objectives were to assess the influence of AR visual cues on axial kinematics, and on the scaling and timing of turning. We hypothesized that AR visual cues would reduce the percent time frozen and the number and duration of FOG episodes compared to the control condition without cues, with no differences compared to the conventional auditory cues. AR visual cues were expected to improve axial kinematics by increasing medial COM deviation, and advancing and increasing head–pelvis separation, compared to both control conditions. Step scaling (measured as step height and its variability) was thought to improve with AR visual cues compared to both control conditions. Turn timing (measured in cadence, peak angular velocity, stride time and its variability, and turn time) was expected to improve compared to the uncued control condition, with no effects compared to the auditory cues.

## Materials and Methods

This study was performed in accordance with the guidelines of the Declaration of Helsinki (1964), was approved by the medical ethics committee Twente (NL66241.044.18), and registered in the Dutch trial registry (NTR7254; 2018-05-28).

### Participant Selection

Sixteen PD patients with a diagnosis of PD according to the UK Brain bank criteria (22) and a subjective experience of FOG on average more than twice a day were included (Table 1). Exclusion criteria were significant cognitive impairment [mini-mental state examination score (MMSE) <24 or frontal assessment battery (FAB) score <13], comorbidity causing severe gait impairments, severe bilateral visual or auditory impairments precluding the participant from using the cues, and an inability to perform a 180° turn unaided. The following questionnaires were taken prior to testing: New Freezing of Gait Questionnaire (NFOG-Q) (23), the MDS-UPDRS part III (24), MMSE (25), and FAB (30). All participants provided written informed consent prior to their inclusion in the study.

**Table 1:** Clinimetrics.

	**Median**	**IQR**
Number of participants	16	
Age (years)	69	13
Gender (% male)	81	
Disease duration (years)	10	9
Years since FOG	4	9
LEDD (mg/day)	1220	776
UPDRS-part III	38	17
Hoehn and Yahr (II/III)	2	1
MMSE	29	2
NFOGQ	18	7
FAB	17	2

*The median and interquartile range (IQR) quartiles are given, unless stated otherwise. FOG, freezing of gait; LEDD, levodopa equivalent daily dose; UPDRS-part III, Unified Parkinson's Disease Rating Scale part III; MMSE, mini-mental state examination (range 0–30); NFOGQ, New Freezing of Gait Questionnaire (range 0–28); FAB, Frontal Assessment Battery (range 0–18). All questionnaires were rated while participants were at the end of a dopaminergic medication cycle (“end-of-dose”)*.

### Experimental Setup

**Figure 1:**
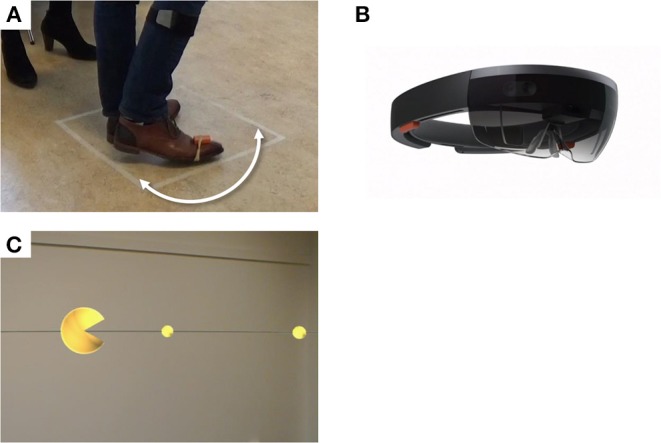
Experimental setup. Participants performed 180° turns around their axes within a 50 × 50 cm square **(A)**, while wearing a HoloLens, a holographic augmented reality headset **(B)**. Trials were performed under the conditions “Visual,” with augmented reality visual cues displayed through the HoloLens **(C)**, “Auditory,” with a metronome beat played at a preferred fixed frequency (not illustrated), and without cues (not illustrated).

A head-mounted AR device, the HoloLens (2017, developer version, Microsoft) (Figure 1B), was used for the holographic display of AR visual cues. The application generating the AR visual cues was custom-built with the game engine Unity (version 2017.1.0f3, Unity Technologies), a software development kit (version 10.0.14393.0, Windows), and Visual Studio (version 14.0.25431.01, Microsoft). Motion data were collected with the MVN Awinda motion capture system (Xsens, Enschede, the Netherlands) (26–28), consisting of 17 inertial measurement units (IMUs) with 3D gyroscopes, accelerometers, and magnetometers (60 Hz sampling frequency, 30 ms latency) attached to the feet (2), lower legs (2), upper legs (2), pelvis (1), hands (2), forearms (2), upper arms (2), sternum (1), shoulders (2), and head (1) with Velcro straps. These motion data were transmitted wirelessly to an experiment laptop with MVN studio 4.4 software installed, and saved for the *post hoc* calculation of kinematic parameters. Two video cameras were directed at the participant from different angles, one directed at the feet and legs and one providing a full-body record. Speakers played the metronome beat, and a beep indicating the start of a trial, at a clearly audible volume. A script built with MATLAB (version 2018a, Mathworks, Inc., Natick, MA, USA) was used to simultaneously play a beep signaling the start of the trial, start the motion capture recording, end the recording after a half turn was fulfilled, and register the timestamps, turning directions and cueing conditions.

### Experimental Procedure

Participants were tested “end-of-dose,” at or shortly after the time they would usually take their (after)noon levodopa and were asked to postpone this levodopa intake until after the experiment. The experimental condition with AR visual cues (“Visual”) consisted of a large yellow sphere displayed with an angle of 6° at 175 cm distance in AR, opening and closing in the turning direction at 2 Hz. The sphere, located in front of the user, moved along with head rotation, thereby “consuming” a series of small yellow spheres displayed with an angle of 2° at 175 cm distance in AR, which were equally spaced at a semicircle around the participant (Figure 1C; Supplementary Video 1). Participants were instructed to rotate their bodies in order to “consume” the small spheres with the large sphere. In one control condition (“Uncued”), no cues were applied. In the second control condition (“Auditory”), participants were encouraged to synchronize their steps to a metronome beat played at a frequency preferred by the participant, determined prior to the measurements. The HoloLens was worn in all conditions, but switched off in the control conditions. The experiment was divided into a training session and two experimental sessions subdivided into three blocks each. The conditions were counterbalanced across each session, with one condition per block. Each block contained 15 trials in which participants performed a 180° turn around their axis within a 50 × 50 cm square taped onto the floor (Figure 1A), as fast as was safely possible (Figure 1A). The turn direction alternated between clockwise and counterclockwise. After the experiment, participants were asked about their previous experiences with cues, AR, and virtual reality (VR), and their experiences with the cues and smart glasses in a structured interview.

### Study Parameters

Parameters for FOG severity were percent time frozen (PTF) and mean number and duration of FOG episodes (29). Axial kinematics were assessed with the maximum medial COM deviation, maximum head–pelvis separation, and time to maximum head–pelvis separation. Spatial and temporal turn parameters were cadence, peak angular velocity, stride time, stride time coefficient of variation (COV), step height, step height COV, and turn time.

### Signal Preprocessing

Data processing was performed with MATLAB (version R2018a, Mathworks, Inc., Natick, MA, USA). Axial kinematic parameters (medial COM deviation and head–pelvis separation) were calculated from the signals of the head and pelvis IMU, and an automated estimation of COM-position outputted by MVN studio 4.4. Signal drift over the course of a recording session was corrected by removal of the linear trend in the orientation signal measured at the start of a trial, and by subtraction of the position at the start of a trial from the estimated COM-position and the pelvis position signals. Medial COM deviation was calculated as the maximum difference between COM position and center-of-pelvis position projected to the inner side of the turn. Maximum head–pelvis separation was defined as the maximum angular difference in orientation of the head and pelvis sensors within the horizontal plane. Footstep-derived parameters (i.e., step height, stride time, and cadence) were calculated from the acceleration and the gyroscope signals of the foot sensors. Foot-ground contacts were detected with a general likelihood ratio test framework (22). FOG episodes were excluded from the calculation of footstep-derived parameters. Step height was calculated as the distance in meters between the ground and the highest vertical foot position during a foot swing. Stride time was defined as the time between two subsequent heel contacts with the same foot. Cadence was defined as the average number of steps per minute.

The number and duration of FOG episodes were scored by two independent raters blinded for the experimental condition from video recordings with the sound switched off. Disagreements were discussed until consensus was reached.

### Statistical Analysis

Statistical analyses were performed with MATLAB R2017b (Mathworks, Inc., Natick, MA, USA; statistics toolbox installed). Alpha was set at 0.05 and adjusted with the Bonferroni–Holmes method for pre-defined *post hoc* planned comparisons (“Visual” vs. “Control', and “Visual” vs. “Auditory”). Extreme outlier values [defined as the values outside 3^*^interquartile range (IQR) below the first or above the third quartile] in kinematic parameters (except for the time to maximum head–pelvis separation) were attributed to technical causes and removed from the analyses. The stride time COV and step height COV were analyzed only in trials with at least three strides. For all parameters, normality of distributions within and across participants were assessed by visual inspection of histograms and Q–Q plots, and tested by Shapiro–Wilk tests. Central tendency within participants was represented by the mean for FOG parameters and the median for kinematic parameters. FOG parameters, maximum medial COM deviation, maximum head–pelvis separation, time to maximum head–pelvis separation, and turn time were non-normally distributed across participants and therefore analyzed with the non-parametric Friedman test and *post-hoc* Wilcoxon-signed rank tests for the effects of cues. The remaining parameters were analyzed with the one-way repeated measures ANOVA and *post-hoc* paired *t*-tests. If Mauchly's test indicated that the assumption of sphericity was violated, *p*-values were corrected with epsilon calculated according to Greenhouse and Geisser. For stride time COV, step height, and step height COV, the analyses were repeated with exclusion of outliers (i.e., values outside 1.5^*^IQR below the first or above the third quartile), because these affected the normality of distributions. We report the *p*-value of the omnibus test (i.e., one-way repeated measures ANOVA or Friedman test) if there was a statistically non-significant group effect. Otherwise, the *p*-values of *post-hoc* pairwise comparisons are reported. Participants who fulfilled the experiment were compared to those finishing a partial experiment with a Fisher's exact test for Hoehn-Yahr stage, Mann–Whitney *U*-tests for other clinimetrics, FOG parameters and non-parametric kinematic parameters, and two-way mixed ANOVAs and a one-way ANOVA for parametric parameters. Consensus on the number and duration of FOG episodes between the two raters was assessed by a Spearman's rank order correlation.

## Results

Twelve participants completed all six blocks, and the four remaining participants finished after three to five blocks because of tiredness or time constraints. Compared to those who completed the entire protocol, participants who performed only a partial experiment had experienced FOG for more years (median 13 vs. 2.5 years) and showed a higher medial COM deviation (mean 0.056 vs. 0.018 m) for all cueing conditions.

One participant was excluded from the analyses of axial kinematics and turn scaling and timing parameters because of technical disturbances of the motion data.

### FOG Parameters

There was a high degree of consensus between raters on the rating of number [*r*s(14) = 0.978, *p* < 0.0005] and total duration of FOG episodes [*r*s(14) = 0.990, *p* < 0.0005] per participant. Fifteen participants experienced FOG at least once throughout the experiment. In those participants who experienced FOG, the mean percent time frozen ranged from 0.4 to 84.2%, with a group mean of 22.3% (all cueing conditions considered together).

**Figure 2:**
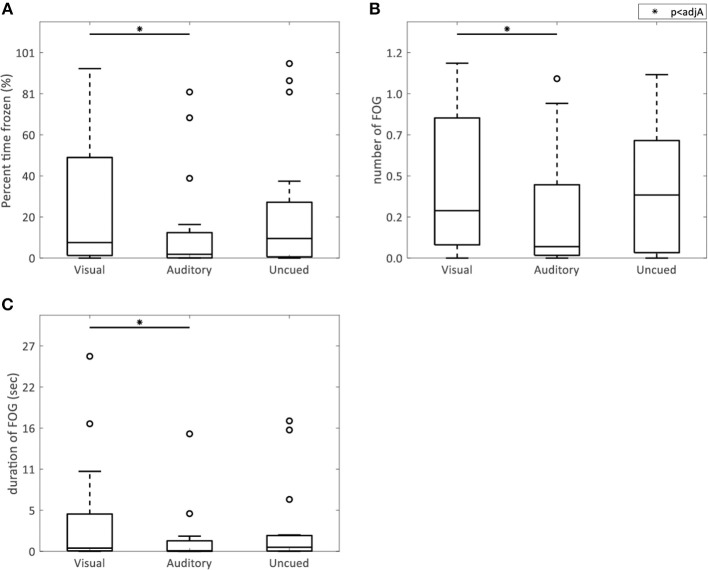
AR visual cues vs. control conditions in FOG parameters. Boxplots showing the percent time spent frozen **(A)**, and the mean number **(B)**, and duration **(C)** of FOG episodes in the conditions with AR visual cues (“Visual”), a metronome (“Auditory”), and no cues (“Uncued”). Significant pairwise comparisons are indicated by horizontal bars with asterisks.

AR visual cues did not significantly alter the percent time frozen (*p* = 0.73) or the mean number (*p* = 0.73) and duration (*p* = 0.78) of FOG episodes compared to the control condition without cues (Figure 2). All FOG parameters were higher with AR visual cues than with auditory cues [percent time frozen (*p* = 0.01), mean number (*p* = 0.02), and duration (*p* = 0.007) of FOG episodes] (Figure 2).

### Axial Kinematics

The AR visual cues significantly increased the time to maximum head–pelvis separation (visual vs. uncued *p* = 0.02; visual vs. auditory *p* = 0.005) (**Figure 4B**), without effect on the maximum head–pelvis separation (*p* = 0.08) (**Figure 4A**) and maximum medial COM deviation (*p* = 0.09) (**Figure 4C**), compared to both control conditions.

### Turn Scaling and Timing

**Figure 3:**
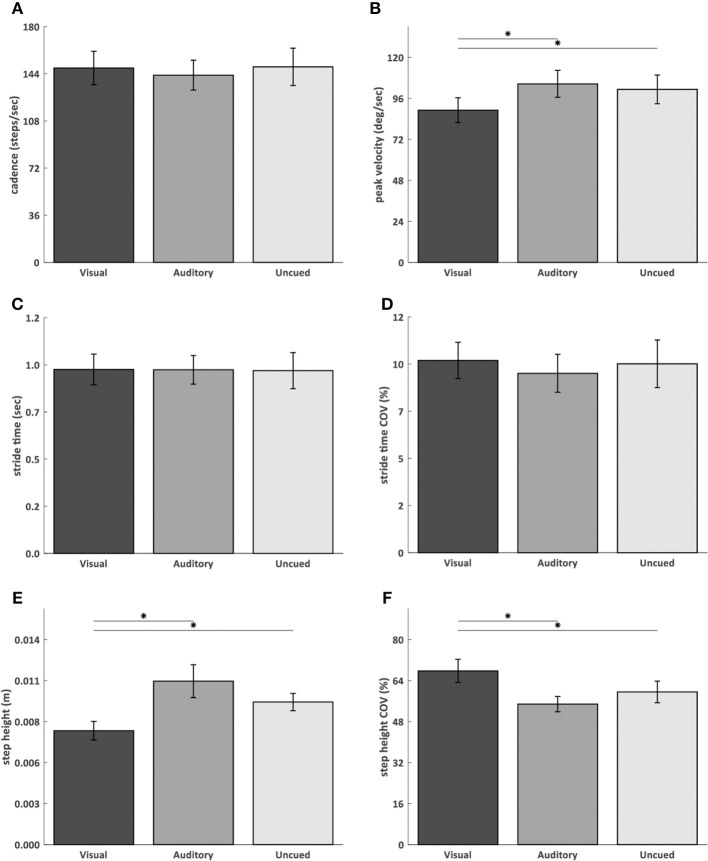
AR visual cues vs. control conditions in normally distributed kinematic parameters. Barplots showing the cadence **(A)**, angular peak velocity **(B)**, stride time **(C)**, stride time coefficient of variation **(D)**, step height **(E)**, and step height coefficient of variation **(F)** in the conditions with AR visual cues (“Visual”), a metronome (“Auditory”) and no cues (“Uncued”). Significant pairwise comparisons are indicated by horizontal bars with asterisks.

**Figure 4:**
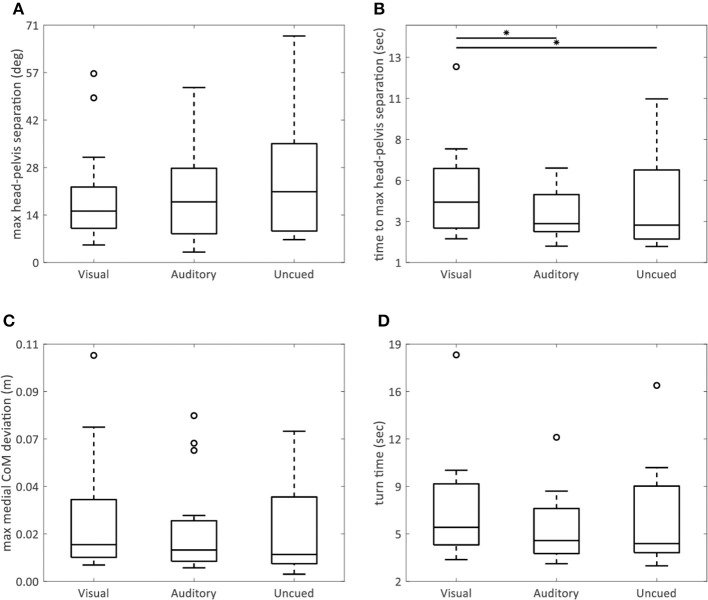
AR visual cues vs. control conditions in non-normally distributed kinematic parameters. Boxplots showing the maximum head–pelvis separation **(A)**, time to maximum head–pelvis separation **(B)**, maximum medial COM deviation **(C)**, and turn time **(D)** in the conditions with AR visual cues (“Visual”), a metronome (“Auditory”), and no cues (“Uncued”). Significant pairwise comparisons are indicated by horizontal bars with asterisks.

AR visual cues significantly decreased peak angular velocity (visual vs. uncued *p* = 0.03; visual vs. auditory *p* = 0.02) (Figure 3B) and step height (visual vs. uncued *p* = 0.02; visual vs. auditory *p* = 0.007) (Figure 3E), and increased step height COV (visual vs. uncued *p* = 0.04; visual vs. auditory *p* = 0.01) (Figure 3F), compared to the auditory and uncued conditions. Cadence (*p* = 0.53) (Figure 3A), stride time (*p* = 0.91) (Figure 3C), stride time COV (*p* = 0.85) (Figure 3D), and turn time (*p* = 0.08) (Figure 4D) were not significantly different between the AR visual cues condition and the control conditions.

Exclusion of outliers did not alter stride time COV, step height, and step height COV.

### User Experience With Cues and Smart Glasses

Most participants (63%) were not accustomed to using cues in the home situation (Supplementary Figure 1A). Six participants used visual cues and five participants used auditory cues, at home (Supplementary Figure 1A). Most participants had never seen an AR (73%) or VR (67%) environment before (Supplementary Figure 1B). When asked about their experience with the AR visual cues, 80% of participants agreed or strongly agreed that the AR visual cues were an easy goal to turn toward, 67% of participants reported that the cues helped direct their attention toward turning, but only a minority (27%) felt that the cues helped them shift their weights (Supplementary Figure 1C). All participants strongly agreed that the color and shape of the visual cues were easy to differentiate, and 87% of participants had no problems localizing the AR visual cues. One participant (7%) indicated that the AR visual cues hindered normal sight, while 40% of participants reported that looking through smart glasses felt different from their normal sight (Supplementary Figure 1C). A minority of participants felt that wearing smart glasses (regardless of cues) was distracting (13%) or restricting (13%), and all but one participant indicated to have easily gotten used to using smart glasses (Supplementary Figure 1C).

## Discussion

We aimed to assess whether AR visual cues improved FOG and turning in place performance in PD patients with FOG. FOG severity, axial kinematics, and turn scaling and timing were compared between an experimental condition with AR visual cues, and two control conditions (metronome and no cues). Contrary to our hypotheses, AR visual cues did not reduce FOG. In fact, FOG was worse with AR visual cues than with the auditory cues, which seemed due to a beneficial effect of the metronome rather than a detrimental effect of AR visual cues on FOG. Also in contradiction with our hypotheses, the AR visual cues worsened some measures of axial kinematics, and turn scaling and timing. We discuss several possible explanations for these findings.

First, stimulating goal-directed movement might by itself be insufficient to improve FOG and turning. Other characteristics of cues, such as their ability to aid in scaling or timing of movement (23), are possibly further prerequisites for cues to be effective. The timing aspect is often provided by auditory cues (8, 17–21), but could also be delivered by visual cues—e.g., by opening and closing the AR visual cues at the preferred stepping speed. To aid in scaling, both the current and targeted foot positions could be represented in AR, thereby providing information on the direction and size of the foot displacement required to reach the target.

Second, the goal provided by the visual cues might have been too distinct from the actual goal of turning. In fact, AR visual cues might have introduced a dual task rather than an integrated turning strategy. The large sphere representing the body position implicitly stimulated body rotation. A more explicit goal, such as discrete targets to step toward, could be more effective. Indeed, a previous study applying transverse strips at a short-circle walkway demonstrated an improvement in FOG, step length, and cadence (24), although these cues not only stimulated goal-directed movement but aided in scaling as well.

Third, wearing smart glasses might have affected turn kinematics. Although wearing comfort of the HoloLens was considerably better than that of previous smart glasses (25), the glasses were still rather heavy. Participants might have kept their heads overly rigid to prevent the glasses from sagging or shifting. This might explain why no effect of cues on head–pelvis separation was found, contrasting earlier work showing a reduction in head–pelvis separation induced by auditory cues (21). Likewise, such increased axial rigidity might have prevented the cues from increasing medial COM shifting. Confirming that smart glasses indeed altered turn kinematics would require a comparison between turning with and without smart glasses.

Fourth, the participants might have been insufficiently familiarized with the smart glasses and cues. Participants were allowed to practice until they felt comfortable with the task and conditions, and all but one participant indicated they easily got used to using the smart glasses. Nevertheless, they might not have mastered using the cues adequately. Indeed, for two-thirds of participants, this was their first encounter with VR or AR, and only a third of participants used cues at home.

A limitation to this study is that participants were not selected for a known cueing responsivity. That two-thirds of our participants were not accustomed to using cues might have been due to unfamiliarity with cues, but also to a previously experienced resistance to cueing effects. Selecting only those patients with a recognized response to cues would increase the potency of experimental cues, but reduce generalizability of the results to patients with an unknown response to cues. A second limitation to this study is the relatively small sample size. The effect size of the AR visual cues might have been smaller than estimated, requiring a larger sample size to detect statistically significant differences.

## Conclusion

The AR visual cues in this study did not improve FOG, and impaired axial kinematics, and turn scaling and timing. Most likely, it takes more than stimulating goal-directed movement to alleviate FOG and improve turning. Whether visual cues delivered through AR earn a place in the repertoire of cueing strategies remains to be established.

## Data Availability Statement

The datasets generated and analyzed during the current study are available in the Donders repository, http://hdl.handle.net/11633/aacths3x.

## Ethics Statement

This study was performed in accordance with the guidelines of the Declaration of Helsinki (1964), approved by the medical ethics committee Twente (NL60687.044.17), and registered in the Dutch trial registry (NTR6409; 2017-02-16). All participants provided written informed consent prior to their inclusion in the study.

## Author Contributions

SJ and JR were involved in the conception and design of the study, the acquisition, analysis, and interpretation of the data, writing of the manuscript, and editing of the final manuscript for submission. HS was involved in the design and building of the AR application. HC was involved in the analysis of the data and critical appraisal of the manuscript. BB critically appraised the manuscript. RW and TH were involved in the conceptual design and setup of this study, the analysis and interpretation of the data, critical revision of the manuscript, and supervision over the study. All authors read and approved the final manuscript.

### Conflict of Interest

The authors declare that the research was conducted in the absence of any commercial or financial relationships that could be construed as a potential conflict of interest. The reviewer GA declared a past co-authorship with one of the authors BB to the handling editor.

## Supplementary Material

Supplementary Figure 1 Answers structured interview. **(A)** Stacked bar plot representing the use of cues by participants in the home-situation. Each horizontal bar represents one participant. The use of multiple cues by one participant is illustrated as multicolored stacked bars. **(B)** Pie charts illustrating how often participants had previously seen an augmented reality (AR, left) or virtual reality (VR, right) environment before. **(C)** Stacked bar plot representing the percentage of answers on a 5-point Likert scale, from “Strongly disagree” to “Strongly agree,” to questions about the experimental cues and smart glasses. All participants fulfilled part **(A,B)** of the structured interview, 15 out of 16 participants fulfilled part **(C)**.

Supplementary Video 1 AR visual cues during turning. Demonstration of the AR visual cues during a turn right followed by a turn left. Here, a hand gesture starts the AR visual cues. During the experiments the cues were started with a remote controller by the researcher, not requiring any action from the participant. Small spheres are equally spaced at a half circle around the participant. Head rotation causes a large sphere to move forwards on a semicircular path, “consuming” the small spheres on its way.

## Abbreviations

FAB: Frontal Assessment Battery
FOG: freezing of gait
MMSE: mini-mental state examination
N-FOGQ: New Freezing of Gait Questionnaire
PD: Parkinson's disease
UPDRS-part III: Unified Parkinson's Disease Rating Scale part III (motor examination)
AR: augmented reality
VR: virtual reality.
