# Supplementary material for: The Effects of Augmented Reality Visual Cues on Turning in Place in Parkinson's Disease Patients With Freezing of Gait
Source: Front Neurol. 2020 Mar 24;11:185. doi: 10.3389/fneur.2020.00185 (PMC7105859; doi:10.3389/fneur.2020.00185)
Supplement: Supplementary Figure 1 — Answers structured interview. (A) Stacked bar plot representing the use of cues by participants in the home-situation. Each horizontal bar represents one participant. The use of multiple cues by one participant is illustrated as multicolored stacked bars. (B) Pie charts illustrating how often participants had previously seen an augmented reality (AR, left) or virtual reality (VR, right) environment before. (C) Stacked bar plot representing the percentage of answers on a 5-point Likert scale, from “Strongly disagree” to “Strongly agree,” to questions about the experimental cues and smart glasses. All participants fulfilled part (A,B) of the structured interview, 15 out of 16 participants fulfilled part (C). [file Data_Sheet_1.DOCX]

## Additional figure 1 Answers structured interview

A

B

C
